# Novel Custom 3D-Printed Glenoid in Reverse Total Shoulder Arthroplasty: A Case Report of Bilateral Total Shoulder Arthroplasties

**DOI:** 10.7759/cureus.78821

**Published:** 2025-02-10

**Authors:** Caroline T Gutowski, Neil Kelly, Catherine J Fedorka

**Affiliations:** 1 Orthopedic Surgery, Cooper Medical School, Rowan University, Camden, USA; 2 Orthopedic Surgery, Cooper Bone and Joint Institute, Cooper University Health Care, Camden, USA

**Keywords:** 3d printed, case report, custom glenoid baseplate, glenoid bone loss, glenoid defect, reverse total shoulder arthroplasty

## Abstract

Glenoid bone loss in the setting of complex reverse total shoulder arthroplasty (rTSA) poses a challenge to surgeons. The advent of custom-made glenoid implants has allowed for the restoration of glenoid alignment and version in cases of significant defects. We present a novel case of a patient who underwent bilateral rTSAs, one of which was performed with a 3D-printed custom glenoid implant. Functional results at one year were comparable between the two shoulders, despite the significant preoperative glenoid defect in the shoulder that required a custom component. This case report and other early results with custom glenoid augments demonstrate promising clinical and radiological outcomes.

## Introduction

Glenoid bone loss in complex reverse total shoulder arthroplasty (rTSA) presents a challenge to surgeons. Addressing significant glenoid bone loss is critical to restore proper function and stability of the shoulder prosthesis and prevent baseplate loosening that can potentially necessitate revision surgery [1]. The utility of three-dimensional (3D) computed tomography (CT) scan planning software to allow surgeons to better define preoperative pathologies and select optimal implants and locations has been previously well-established [2]. In the setting of significant glenoid bone stock defects, however, off-the-shelf augments and wedges may be inadequate. In these cases of significant bone loss, custom-made implants and structural bone grafts may be considered, with the latter being associated with increased failure rates [3-5]. Thus, the advent of 3D-printed, custom glenoid baseplates has allowed for the restoration of glenoid alignment and version when premade glenoid baseplates prove inadequate [4,6-11]. While there are limited large-sample studies investigating the role of patient-specific glenoid augments, early results demonstrate promising clinical outcomes in cases of large glenoid bone loss, both in primary and revision shoulder arthroplasty [4,8,10-12].

We present a unique case of bilateral rTSAs, the right with anatomy appropriate for a standard augmented baseplate and the left with a significant glenoid bone defect warranting treatment with a custom-made prosthesis. Unlike other case reports currently in literature, this case specifically allows us to use the contralateral shoulder, with a standard glenoid augment, as a control. The patient provided consent for the submission and publication of data involving this case.

## Case presentation

**Figure 1:**
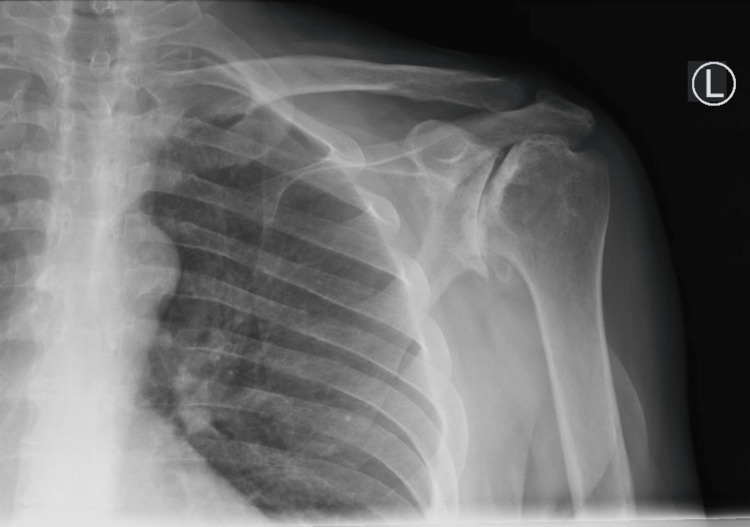
Preoperative X-rays of the left shoulder The image depicts a Grashey view of the left shoulder

**Figure 2:**
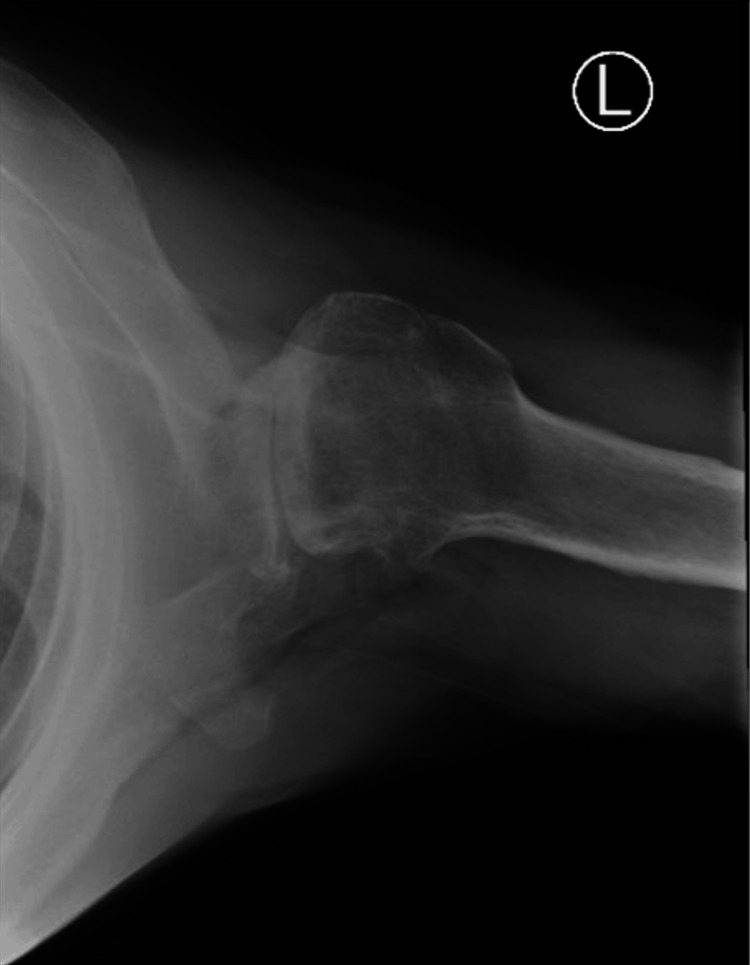
Preoperative X-rays of the left shoulder The image depicts an axillary view of the left shoulder

**Figure 3:**
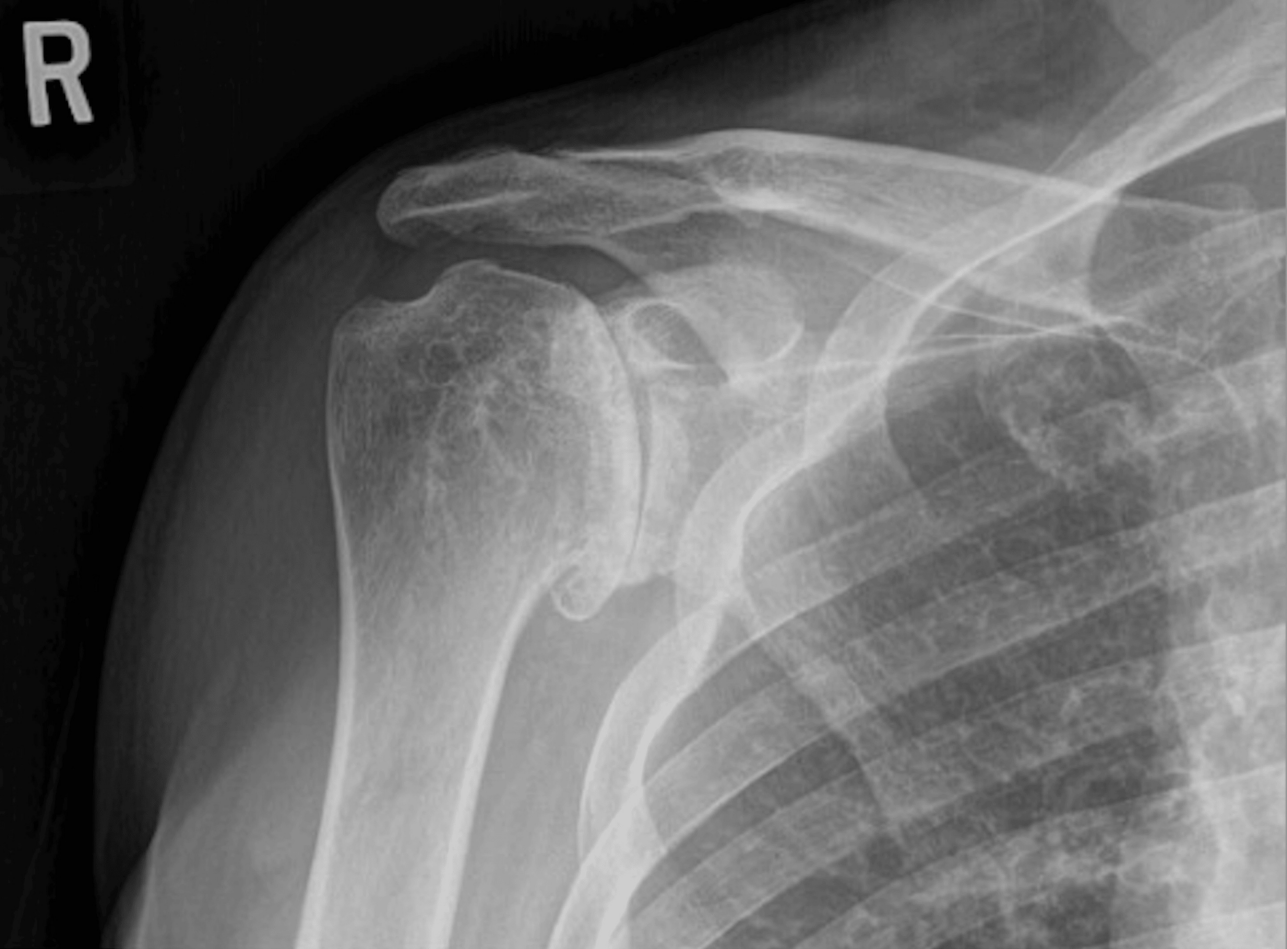
Preoperative X-rays of the right shoulder The image depicts a Grashey view of the right shoulder

**Figure 4:**
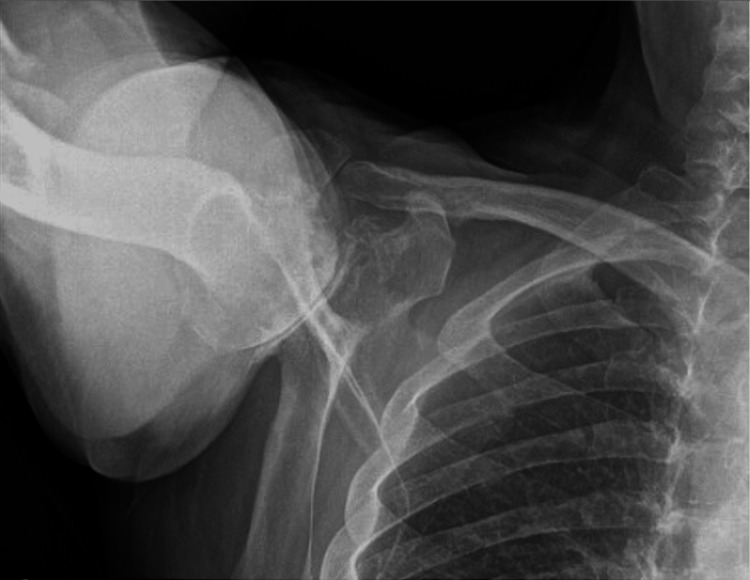
Preoperative X-rays of the right shoulder The image depicts an attempted axillary view of the right shoulder

A 62-year-old right-hand dominant man was evaluated in the clinic for chronic, bilateral shoulder pain. The patient’s relevant past medical history included cervical radiculitis, cervical spondylosis without myelopathy, benign prostatic hyperplasia, hyperlipidemia, obstructive sleep apnea, and gastroesophageal reflux disease. His past surgical history included a left shoulder arthroscopy (30 years ago) and left hernia repair. The patient reported tobacco use with a 45-pack-year history. The patient reported a three-year history of bilateral aching, burning, and constant pain located over his shoulders with radiation to his elbow. The patient reported 10/10 pain even at rest that wakes him from sleep and is further aggravated by overhead motion. His activities of daily living have been severely limited by his shoulder pain, which he described as so severe that he can barely dress himself. He has a history of treatment with corticosteroid injections in bilateral shoulders, which provided some relief to the left shoulder but did not improve his right shoulder pain. Preoperative radiographs were obtained demonstrating severe glenohumeral arthritis bilaterally (Figures 1-4).

**Figure 5:**
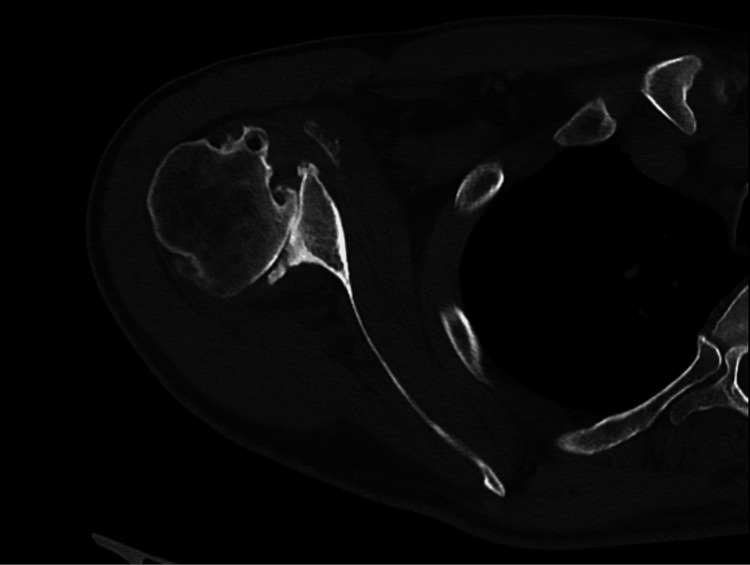
Preoperative CT of the right shoulder CT: computed tomography The image depicts an axial view of the right shoulder

**Figure 6:**
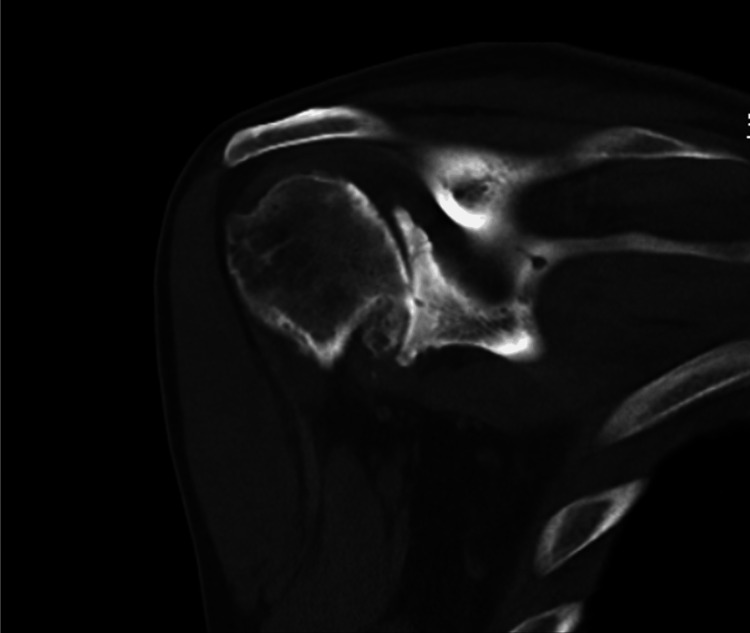
Preoperative CT of the right shoulder CT: computed tomography The image depicts a coronal view of the right shoulder

**Figure 7:**
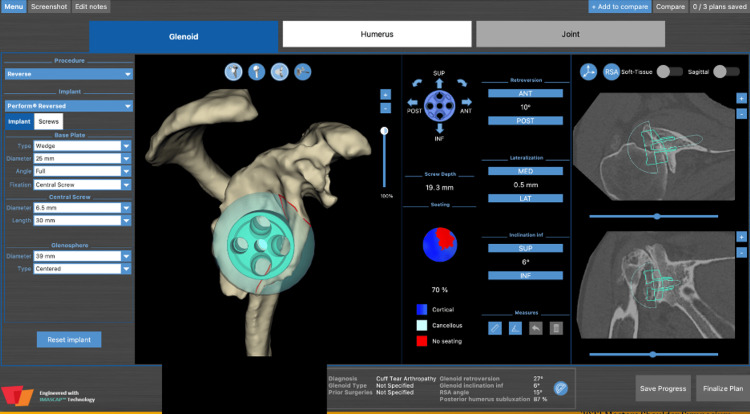
Preoperative planning for right rTSA rTSA: reverse total shoulder arthroplasty

A CT scan was performed for preoperative planning and 3D planning software (Blueprint, Stryker, Mahwah, NJ) was utilized for the right shoulder (Figures 5, 6). The patient’s preoperative retroversion was measured to be 27° with 6° of inferior inclination. Given the severity of his deformity, rTSA was planned to involve a full-wedge augment to correct retroversion back to 10° (Figure 7). A custom 3D-printed glenoid could not be utilized as the deformity was too large for the maximum envelope allowed for this particular implant. 

**Figure 8:**
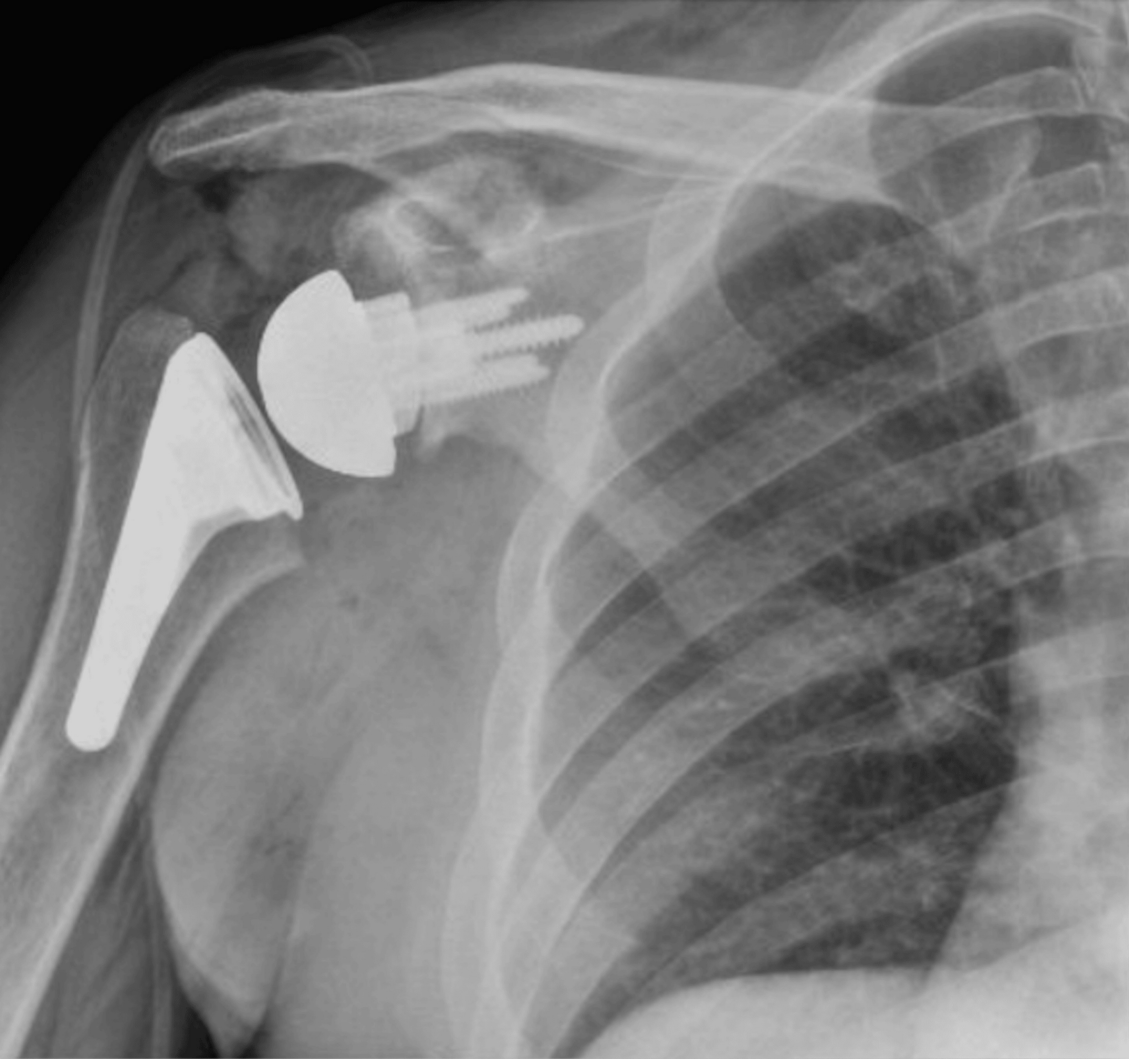
Postoperative X-rays of the right shoulder following reverse shoulder arthroplasty The image depicts an anteroposterior (AP) view of the right shoulder

**Figure 9:**
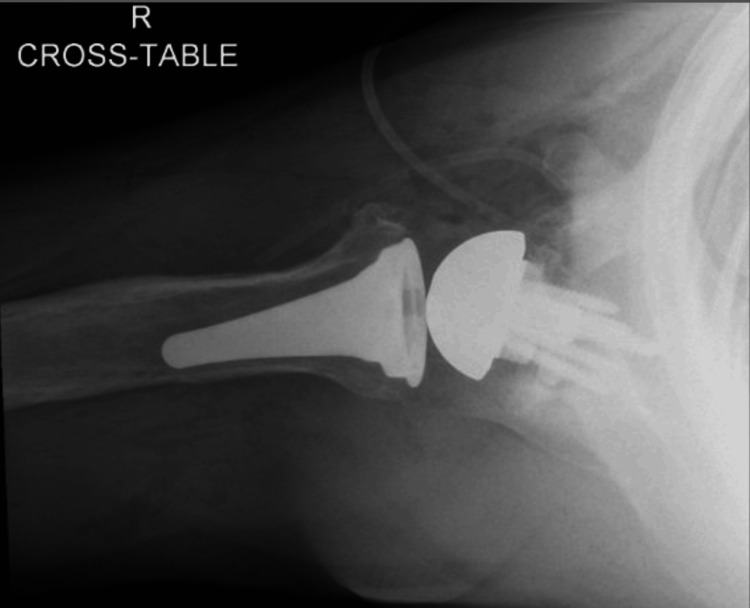
Postoperative X-rays of the right shoulder following reverse shoulder arthroplasty The image depicts an axillary view of the right shoulder

The patient was placed in a beach chair position, and a standard deltopectoral approach was used. The subscapularis was partially torn, but the remainder was peeled off the lesser tuberosity, and the humeral head was dislocated. A humeral head cut was performed at 30° of retroversion and 135° of inclination using an intramedullary guide. Attention was then turned to the glenoid. An anterior and inferior capsular release was performed. A custom guide was used to aid in targeting for reaming and central screw placement. The glenoid was then prepared according to the manufacturer’s technique with gentle reaming anteriorly to correct some retroversion and allow seating of the baseplate. A 25 mm full augment baseplate was utilized to correct retroversion to 10°. A 39 mm standard glenosphere was placed. The humerus was prepared according to the manufacturer’s technique. After trialing implant sizes and assessing for tension and stability, the final size 3 stem and 3 mm polyethylene insert were impacted, and the shoulder was reduced. The subscapularis was deemed irreparable and the biceps was tenodesed to the pectoralis. Final radiographs in the operating room indicated stable positioning of the implants (Figures 8, 9). The time from incision to surgery end was 132 minutes. The patient was placed in a simple sling and instructed to be non-weight-bearing (NWB) on the right upper extremity with permission to do passive range-of-motion (ROM) exercises to forward flexion (FF) of 120° and external rotation (ER) of 20°.

At a two-week follow-up, the patient reported doing well with a 0/10 pain score. The sling was discontinued and a prescription to begin physical therapy (PT) was provided. At six weeks postoperatively, the patient reported no pain. Active ROM (AROM) for FF, abduction, and ER measured 130°, 90°, and 20°, respectively. The plan for the right shoulder was to continue with PT for shoulder ROM but avoid strengthening exercises for the time being. At three months after the right rTSA, the patient reported doing well. AROM for FF, abduction, ER, and internal rotation (IR) increased to 160°, 90°, 50°, and L5, respectively. 

**Figure 10:**
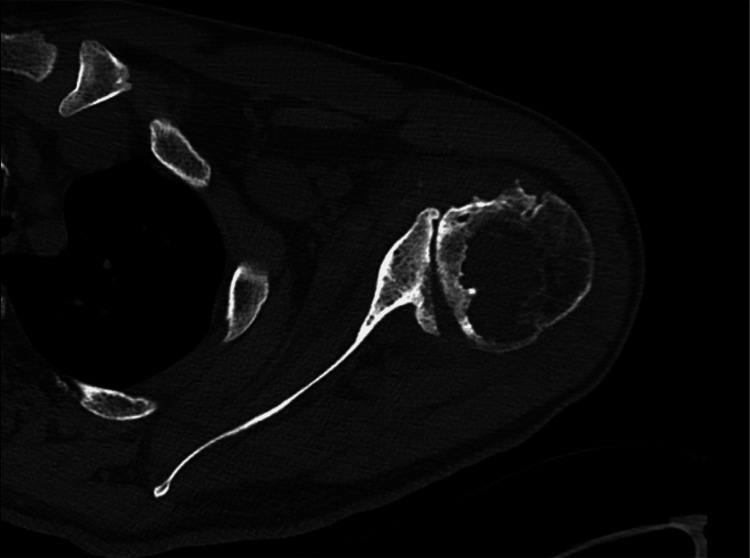
Preoperative CT of the left shoulder CT: computed tomography The image depicts an axial view of the right shoulder

**Figure 11:**
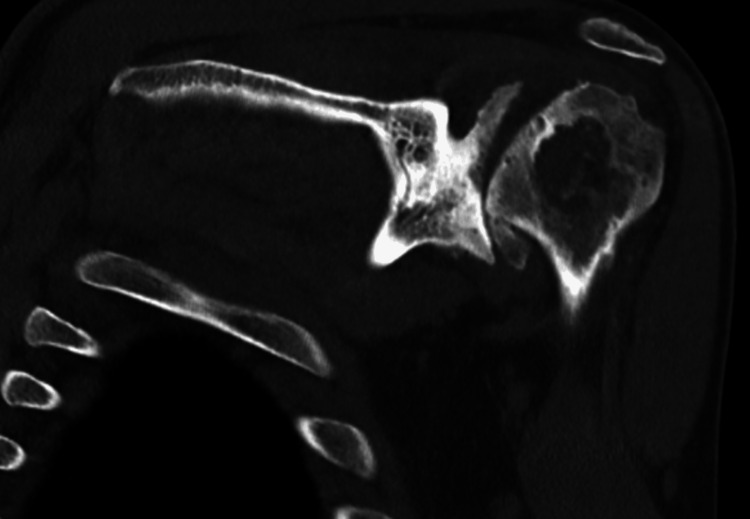
Preoperative CT of the left shoulder CT: computed tomography The image depicts a coronal view of the right shoulder

The patient expressed a desire to proceed with rTSA of the left shoulder. Previous radiographs of the left shoulder demonstrated severe glenohumeral osteoarthritis with a large subcortical cyst in the humeral head. Due to severe posterior glenoid remodeling and dysplasia with posterior humeral head subluxation, a CT scan was ordered to plan for the evaluation of glenoid bone stock (Figures 10, 11). Imaging demonstrated a retroverted glenoid consistent with a B3 glenoid according to the Walch criteria. The B3 glenoid as defined by Walch is monoconcave, where expansion of the neoglenoid wear has eroded anteriorly to create 15° of retroversion and ≥70% posterior humeral subluxation [13-15].

At the preoperative visit for the left rTSA, the patient reported 6/10 pain in the left shoulder. Left shoulder AROM for FF, abduction, and ER measured 90°, 80°, and 20°, respectively. The patient elected to proceed with a left rTSA, with surgery planned for four months after the right rTSA operation.

**Figure 12:**
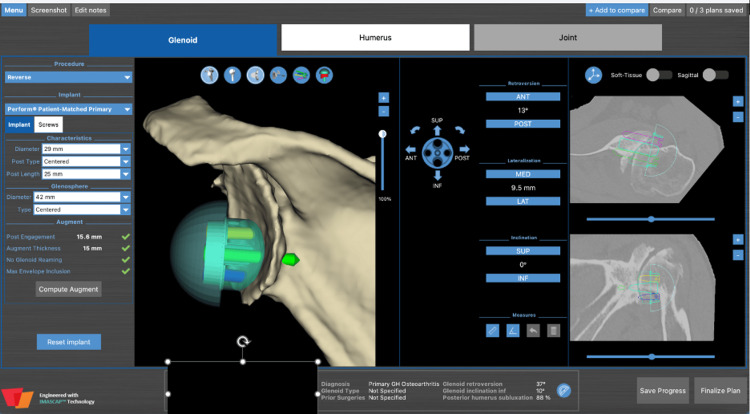
Preoperative planning for left rTSA with custom 3D printed glenoid implant rTSA: reverse total shoulder arthroplasty; 3D: three dimensional

CT scan was utilized for preoperative planning with the same 3D planning software (Figure 12). The patient’s preoperative retroversion measured 37° with 17° of inferior inclination. Given the severity of his deformity, rTSA with a custom 3D-printed glenoid was planned (Shoulder ID, Stryker Corporation, Mahwah, NJ) (Figure 12). Custom implants are often used when there is a significant glenoid deformity, such as an extreme version (>20°) or inclination. These custom implants can be beneficial when standard implants, including augmented implants, cannot be implanted with <10° of retroversion, neutral or inferior inclination, or require excessive reaming to achieve enough backside seating. This specific custom implant corrected the glenoid to 13° of retroversion with 0° of inclination. 

**Figure 13:**
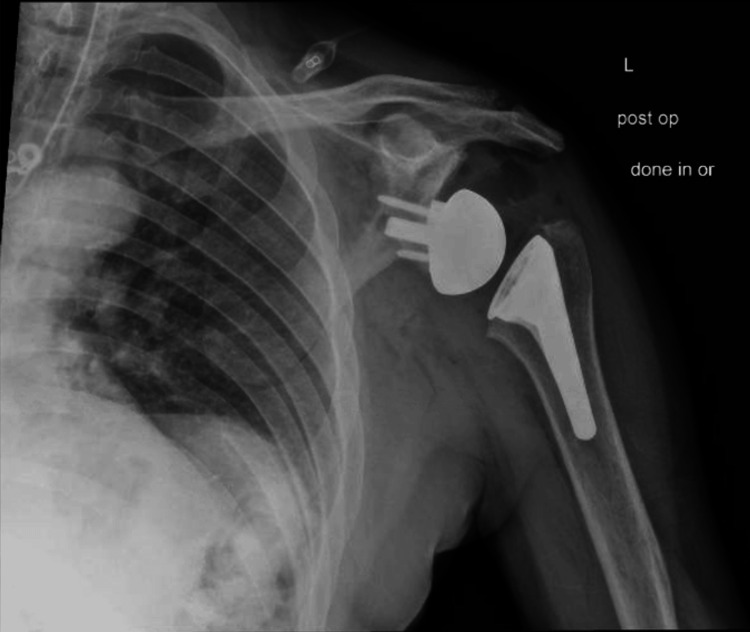
Postoperative X-rays of the left shoulder following rTSA with custom glenoid implant rTSA: reverse total shoulder arthroplasty; AP: anteroposterior The image depicts an AP view of the right shoulder

**Figure 14:**
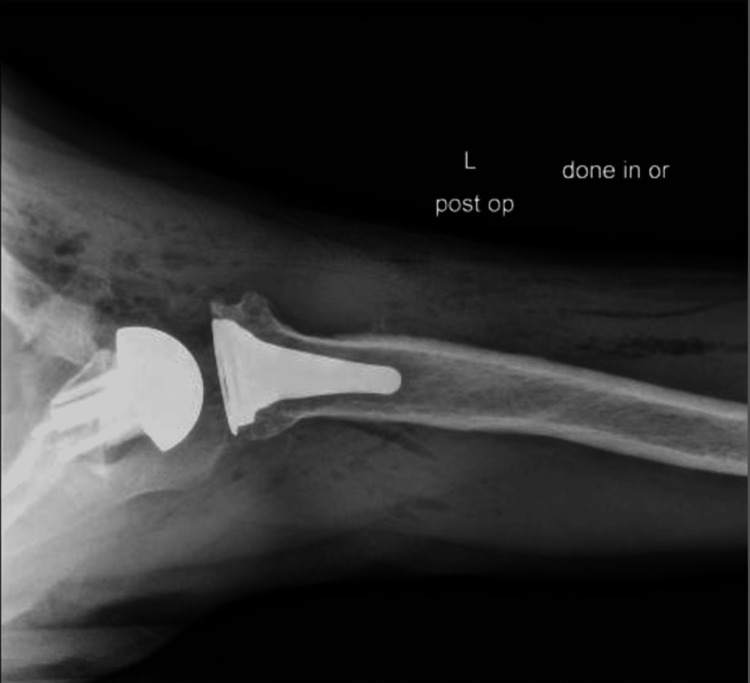
Postoperative X-rays of the left shoulder following rTSA with custom glenoid implant rTSA: reverse total shoulder arthroplasty The image depicts an axillary view of the right shoulder

For the left shoulder, the patient was again placed in a beach chair position, and a standard deltopectoral approach was used. The same approach and technique for exposure were used as that of the right shoulder. Once the humeral head cut was performed, the glenoid was exposed. Any remaining cartilage was removed with a scraper, with care to not remove any bone. A custom guide was utilized to drill the center pin. An anti-rotation pin was then placed. As this is a custom implant, no reaming is performed. The glenoid was kept intact without reaming or removing osteophytes to ensure proper placement of the guide. The center hole for the central post is drilled at the correct depth. The custom glenoid was then impacted into place using the anti-rotation pin to guide the correct orientation. The peripheral screws were then drilled and placed. A 42 mm glenosphere was utilized. Attention was then turned back to the humerus which was again prepared according to the manufacturer’s technique. Final implants for the humeral portion included a size 3 stem with a 6 mm polyethylene. The subscapularis was irreparable, and the biceps was tenodesed to the pectoralis. Final X-rays in the operating room demonstrated stable implants and a reduced shoulder (Figures 13, 14). The time from incision to procedure end was 138 minutes. The patient was placed in a sling and was instructed to be NWB in the left upper extremity with permission to perform passive ROM with FF to 120° and ER to 20°.

At two weeks post-left rTSA, the patient reported improvement in pain compared to preoperative levels. The patient had normal motor and sensory responses in the axillary, musculocutaneous, median, radial, and ulnar nerve distributions. Left AROM for FF and abduction measured 60° and 70°, respectively. The plan was to begin PT to work on motion and progress toward AROM between four and six weeks.

At six weeks follow-up for left rTSA, the patient reported no pain. AROM and passive ROM (PROM) of the left shoulder for FF, abduction, and ER both measured 150°, 100°, and 30°, respectively. At the same visit, AROM and PROM for the right shoulder measured 160°, 100°, and 30°, respectively.

At three months post-left rTSA and seven months post-right rTSA, the patient reported being pain-free in both shoulders with good functional motion. AROM for the left shoulder for FF, abduction, ER, and IR measured 150°, 90°, 50°, and L5, respectively. For the right shoulder, AROM for FF, abduction, ER, and IR measured to 150°, 90°, 50°, and L5, respectively. The strength of abduction, ER, and IR was 4/5 bilaterally.

**Figure 15:**
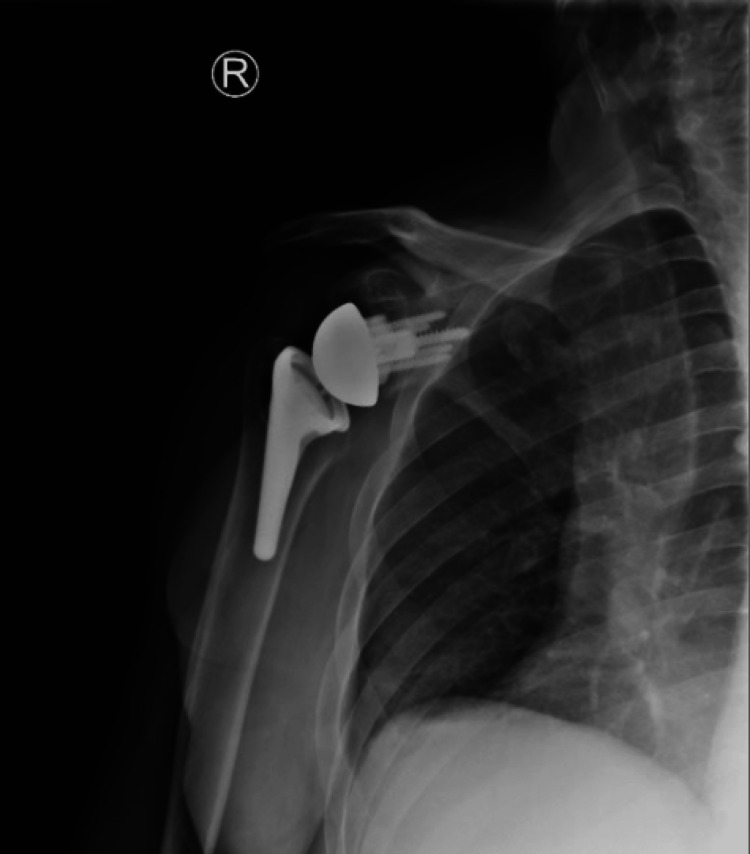
Postoperative X-rays of the right shoulder at one-year follow-up AP: anteroposterior The image depicts an AP view of the right shoulder

**Figure 16:**
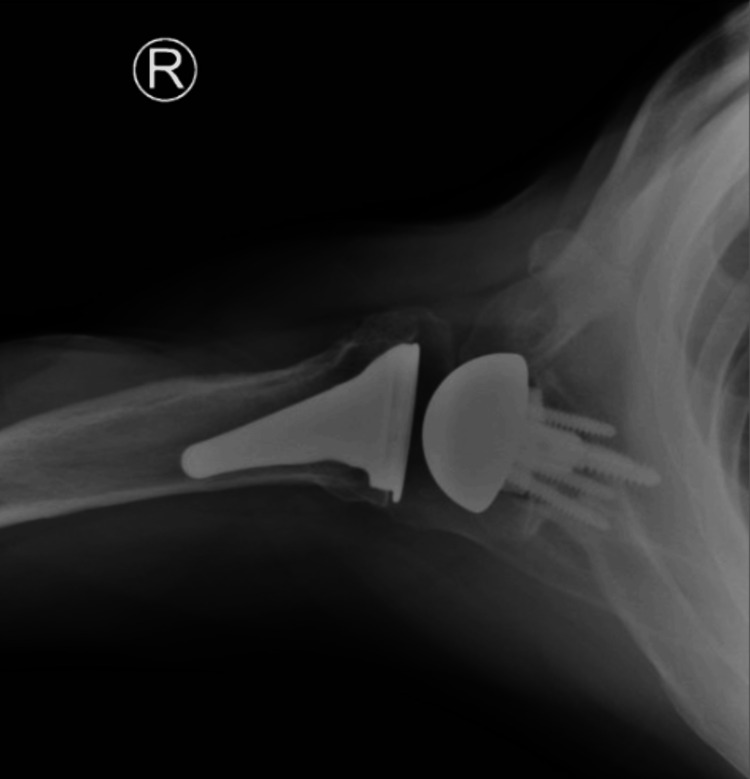
Postoperative X-rays of the right shoulder at one-year follow-up The image depicts an axillary view of the right shoulder

At nine months follow-up for left rTSA, AROM in the left shoulder for FF, abduction, ER, and IR was 150°, 90°, 60°, and L5, respectively. At a one-year postoperative visit for the right rTSA, AROM for FF, abduction, ER, and IR measured 150°, 90°, 50°, and L5, respectively. The one-year postoperative American Shoulder and Elbow Surgeons (ASES) shoulder score for the right shoulder was 95. Figures 15-16 depict X-rays demonstrating a stable rTSA at this point. 

**Figure 17:**
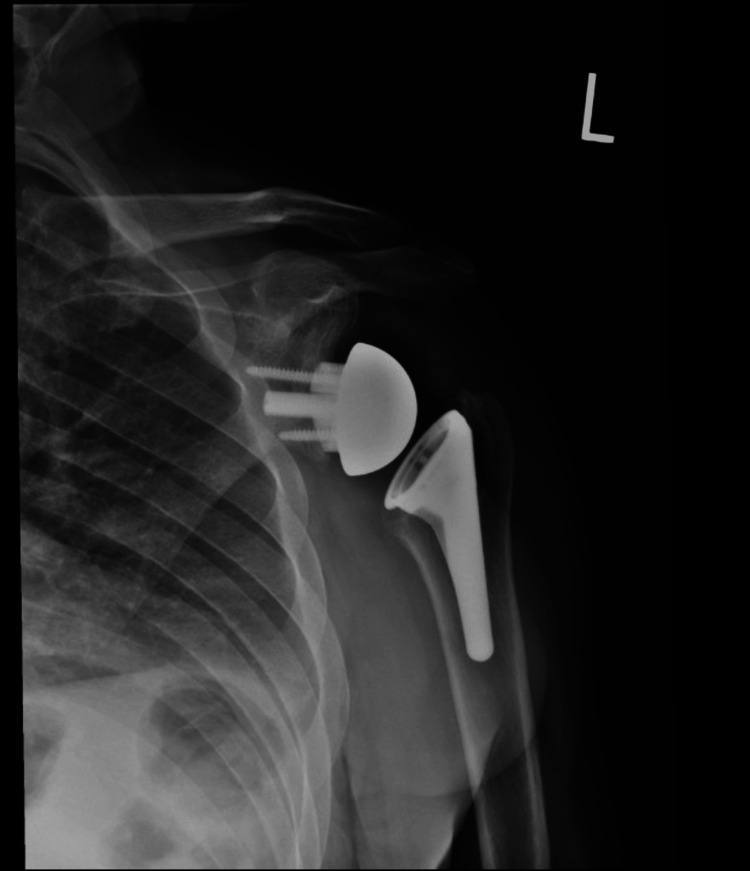
Postoperative X-rays of the right shoulder at one-year follow-up AP: anteroposterior The image depicts an AP view of the right shoulder

**Figure 18:**
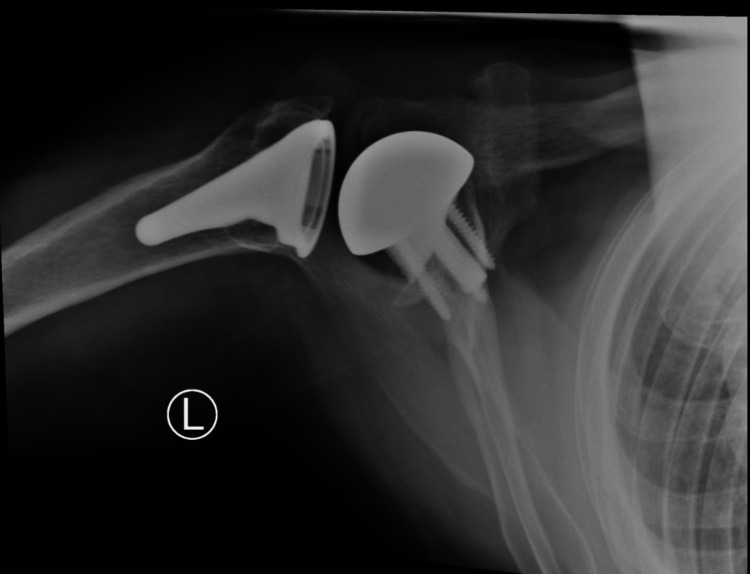
Postoperative X-rays of the right shoulder at one-year follow-up The image depicts an axillary view of the right shoulder

At one-year follow-up for the left rTSA and 16-month follow-up for the right rTSA, AROM in both the left and right shoulders for FF, abduction, ER, and IR was 150°, 90°, 30°, and L5, respectively. No complications arose following the left and right rTSAs. The one-year postoperative ASES shoulder score for the left shoulder was 93. Radiographs at this visit showed no evidence of hardware failure or loosening (Figures 17, 18).

## Discussion

Herein, we present a case of a patient who underwent bilateral rTSAs, one of which was performed with a 3D-printed custom glenoid implant. This case report demonstrates the effective use of a novel custom patient-matched glenoid augment with rTSA in the setting of significant defects for which premade augments and wedges were inadequate. On the left side, the extreme glenoid version (>20°) did not accommodate a standard augmented implant without excessive retroversion and reaming; thus, the custom implant was indicated. Utilizing the 3D-printed implant allowed the surgeon to preserve bone while trying to restore anatomy. In our patient, the custom implant allowed for greater AROM at the six-week postoperative visit, compared to the right at six weeks postoperatively; however, these equalized over time. AROM and one-year ASES scores in both shoulders at one-year follow-up were comparable, despite the significant preoperative glenoid defects in the left shoulder. 

Glenoid component failure is a relevant complication from rTSA, secondary to malposition and lack of implant purchase into adequate bone stock [1,2,16-20]. The use of patient-specific instrumentation (PSI) with 3D-printed guides has been well-reported in the literature, demonstrating more accurate glenoid component placement and longer superior and inferior screw placement [1,2,16,18-21]. On the right rTSA in our patient, a custom cutting guide was used to aid in targeting for reaming and screw placement. While custom PSI guides improve baseplate fixation, they do not address component failure secondary to severe glenoid bone loss. 

Strategies to address glenoid defects include bone grafting, alternate center line, augmented glenoid components, and the more recent innovation, custom glenoid implants [3]. Structural bone grafting, either allograft or autograft, addresses severe glenoid defects but is not without limitations including demanding surgical technique, higher rates of infection, and bone resorption leading to loosening and failure [3,10,22]. The alternate central line requires anteversion and interior tilting of the central baseplate into the column of bone parallel to the scapular spine, which is typically preserved in cases of severe glenoid bone loss [3,23]. This technique prioritizes glenoid baseplate fixation over anatomic placement of the glenoid, which can negatively impact functionality, particularly internal rotation, and has been associated with higher rates of scapular spine fractures and scapular notching [23]. Augmented glenoid components can restore the anatomic joint line and minimize nonunion risk; however, these implants are restricted by their design. Augments typically include fixed angle full or half wedge implants that cannot accommodate the glenoid defect, as seen in our patient [3,10]. Compared to these techniques, custom-made prostheses provide defect-specific shapes and thus suitability for unique bone loss patterns [9].

An advantage of this particular implant is the ability for the surgeon to individually plan the custom glenoid baseplate, rather than have an engineer design it. The plan is then approved by engineers, and the implant is 3D-printed. Custom-made prostheses offer defect-specific shapes and hence suitability for extensive bone loss; however, disadvantages include increased cost compared to conventional implants and the potential for greater shearing forces due to the increased distance between the center of rotation and the bone/metal line [9]. The custom implant utilized in this case is another option to be considered in patients with glenoid pathology that standard baseplates may not accommodate without significant reaming. 

Recovery in the ROM and function in the left shoulder of our patient supports the utility of computer-aided designs and 3D-printed models for the reconstruction of significant glenoid defects. At one-year follow-up from rTSA with the custom glenoid augment, our patient achieved 150° of FF and 90° of abduction, similar to ROM reported in the current literature which ranges from 105° to 160° and 45° to 70° for FF and abduction, respectively [4,6,8,9]. CT with virtual preoperative planning software and novel 3D printing technology has led to the manufacture of custom-made implants [7]. These custom implants allow surgeons to define preoperative pathology and optimize implant location and design while also offering a ream-free, bone-preserving technique to minimize loss of glenoid bone stock [3,4,8,9]. While data is limited by the recent introduction of these implants, this case report and other early results are promising [4,5,8,10-12,16]. Future studies should focus on outcomes in larger samples of patients and developing a classification system that allows surgeons to design custom implants based on bone loss morphology and extension.

## Conclusions

Complex glenoid bone loss in rTSA poses a significant challenge to surgeons, as off-the-shelf glenoid augments and wedges are often inadequate to address the defect. This case report demonstrates the efficacy of a new 3D-printed glenoid component for rTSA to address severe glenoid bone loss. This case specifically allows us to use the contralateral shoulder, where a standard augmented baseplate was utilized, as a reliable comparison. Despite the significant preoperative glenoid bone loss in the left shoulder, both shoulders achieved comparable AROM and ASES scores at one-year follow-up. Final active abduction and FF were similar to values reported in the existing literature on rTSA with custom glenoid augments. While literature is limited by the recent advent of these implants, early results with custom glenoid components have demonstrated significant clinical improvements in patients with complex glenoid defects.
